# Primary cilia have a length-dependent persistence length

**DOI:** 10.1007/s10237-019-01220-7

**Published:** 2019-09-09

**Authors:** Justin Flaherty, Zhe Feng, Zhangli Peng, Y.-N. Young, Andrew Resnick

**Affiliations:** 1 Department of Physics, The Ohio State University, Columbus, USA; 2 Department of Aerospace and Mechanical Engineering, University of Notre Dame, Notre Dame, IN USA; 3 Department of Mathematical Sciences, New Jersey Institute of Technology, Newark, NJ 07102 USA; 4 Department of Physics, Center for Gene Regulation in Health and Disease, Cleveland State University, Cleveland, OH USA; 5 Department of Bioengineering, University of Illinois at Chicago, 851 S Morgan St, Chicago, IL 60607 USA

**Keywords:** Primary cilia, Elastic shell, Mechanobiology

## Abstract

The fluctuating position of an optically trapped cilium tip under untreated and Taxol-treated conditions was used to characterize mechanical properties of the cilium axoneme and its basal body by combining experimental, analytical, and computational tools. We provide, for the first time, evidence that the persistence length of a ciliary axoneme is length-dependent; longer cilia are stiffer than shorter cilia. We demonstrate that this apparent length dependence can be understood by a combination of modeling axonemal microtubules as anisotropic elastic shells and including actomyosin-driven stochastic basal body motion. Our results also demonstrate the possibility of using observable ciliary dynamics to probe interior cytoskeletal dynamics. It is hoped that our improved characterization of cilia will result in deeper understanding of the biological function of cellular flow sensing by this organelle.

## Introduction

Primary cilia are slender hair-like structures, several microns long and 0.2 $\upmu$m in diameter, present on most vertebrate cells, that protrude from the cell body into the extracellular space. Primary cilium axoneme structure consists of 9 microtubule doublets anchored in the basal body, which itself is a highly organized structure comprising a centrosome (microtubule triplets), transition fibers, a rootlet, and the basal foot (Garcia and Reiter 2016). Demonstrations that bending a primary cilium via fluid flow (Jin et al. 2013), optical tweezers (Resnick and Biomed 2010), or micropipette (Praetorius and Spring 2001) initiates intracellular calcium release imply that physiologically, the primary cilium is a flow sensor. However, the biological significance of this function remains unclear in part due to incomplete understanding of the dynamics of the cilium in the presence of flow (Lin et al. 2003; Ma et al. 2013; Delling et al. 2016). We hypothesize that the mechanosensing function of cilia can occur by straining microtubule structural elements in a similar manner to actin-mediated mechanosensation (Mofrad and Kamm 2010). Indeed, preliminary results indicate that the basal body may have a role in differentiating mechanosensation from chemosensation (Jin et al. 2013; Hu and Nelson 2011; Lin et al. 2013).

Because bending the cilium involves mechanical stress and strain, one of our research foci centers on characterizing the mechanical properties of the primary cilium and exploring ways to modify the flow response by pharmacologically altering the mechanical properties of the primary cilium. Historically, cilia have been modeled as homogeneous cantilevered (Euler–Bernoulli) beams (Schwartz et al. 1997), and reported measurements of the flexural rigidity ‘*EI*’ vary widely (Downs et al. 2014).

Because ciliary axonemes are composed of microtubule doublets, we quickly examine what is known about microtubule mechanics. A longstanding and considerable disagreement over reported values of microtubule flexural rigidity (or persistence length $l_{\mathrm{p}}$) is beginning to be resolved by treating microtubules as transversely isotropic shells rather than either homogeneous cylinders or isotropic shells (Liu et al. 2012; Tuszynski et al. 2005; Pampaloni et al. 2006; Memet et al. 2018; Gao et al. 2010). We therefore expect that the mechanical response of cilia also cannot be modeled in terms of a homogeneous cylinder or collection of isotropic shells. In this report, we present evidence for modeling cilia as an independent (mechanically uncoupled) collection of transversely isotropic tubes and then discuss the effect of low concentrations of Taxol on ciliary mechanics.

We performed experiments that probe the mechanical response of primary cilia in a low-strain regime, corresponding to thermal fluctuations, by measuring stochastic fluctuations of an optically trapped cilium tip as a function of the cilium length. High-strain deformations are associated with, for example, buckling (Memet et al. 2018); it is unclear how our results would extrapolate to nonlinear deformations. By applying an optical trap to the distal end of a bare cilium and tracking the fluctuating position of the distal end, we constructed a structural model of a primary cilium that accounts for both axonemal mechanics and basal attachment via the basal body. Crucially, we demonstrate that like single microtubules, the primary cilium also displays a length-dependent persistence length $l_{{\rm p}}(L)$, and a structural model based on anisotropic (rather than isotropic) shells provides quantitative agreement with our data. Our model also provides best-fit estimates for fluctuations of the basal body which agree with the few existing measurements. We also performed experiments probing the mechanical response of cilia in the presence of low concentrations of Taxol, a molecule well-known to alter mechanical properties of microtubules.

One important motivation for the work presented here is the known cellular autoregulation of the cilium length. While the molecular mechanism is generally understood (Marshall and Rosenbaum 2001), the function of length regulation is not. Because deformation of a cilium is associated with flow sensing, a length-dependent persistence length could imply that the length of a cilium is related to the sensitivity of flow sensing, and so length regulation could be a mechanism to regulate either sensor setpoints or sensor sensitivity.

## Materials and methods

### Epithelial cell culture

Our experimental protocols for the growth, maintenance, and pharmacological manipulations of ciliated epithelial cells have been published elsewhere (Resnick 2015, 2016; Glaser et al. 2014), so here we only provide a brief summary. Low-passage number Madin–Darby Canine Kidney (MDCK, ATCC) cells were grown to confluence in 60-mm-diameter plastic petri dishes at 37 $^{\circ }\hbox {C}$ and allowed to differentiate for 72 h before trapping. Differentiation conditions were established by reducing the concentration of fetal bovine serum (FBS) from 10 to 1.5%. A confluent epithelial tissue monolayer was then placed within our temperature controlled microscope sample incubation chamber (Solent scientific) for imaging and trapping.

### Optical trapping

Our experimental protocols for the optical trapping of cilia have been published elsewhere (Resnick 2015, 2016; Glaser et al. 2014), so here we only provide a brief summary.

Trapping was carried out with the cells held at 37 $^{\circ }\hbox {C}$. We used an upright Leica DM6000B microscope to perform imaging and trapping of cilia. The microscope objective used for the experiments was a 63X NA 0.90 dipping objective designed to be directly immersed into the culture media. This trapping geometry is known as a single-beam gradient trap, as a single beam is sufficient to confine the trapped object in all three dimensions. The trapping laser was a 0.5 W diode-pumped Nd:YAG continuous-wave single-mode laser (CrystaLaser CL1047-500) aligned to the optical axis of the microscope using a 5-degree of freedom (*x* axis, *y* axis, *z* axis, pitch, and yaw) mount. The laser beam was expanded to fill the back aperture of the objective lens and entered the microscope through a side port at the fluorescence cube turret. A side-looking dichroic mirror (Chroma) directed the trapping beam down onto the sample, and use of a dichroic allowed unimpeded, simultaneous, viewing of the trapped cilium with brightfield imaging. For our trap, the beam waist is 0.3 $\upmu$m and Rayleigh length is 0.4 $\upmu$m. The forward scattered trapping light was detected by a quadrant photodiode (QPD). The position of the cilium tip was sampled by the QPD and digitally acquired by a custom LabVIEW routine at a rate of 50 kHz.

### Trapping cilia

A cultured monolayer of cells was placed within the microscope sample holder for trapping. The culture was first washed three times with DMEM to remove floating cell debris which could fall into the trap, and the media was replaced with HEPES-buffered DMEM (pH 7.46) without phenol red, which would fluorescence under the trapping beam.

Although we disposed of cell cultures after trapping measurements, the cultures tolerate trapping conditions well. The trapping beam does not appear to damage our cells (Leitz et al. 2002; Neuman et al. 1999). Cultures typically survive several hours and recover if the DMEM is replaced by culture media and cultures returned to the incubator.

**Fig. 1:**
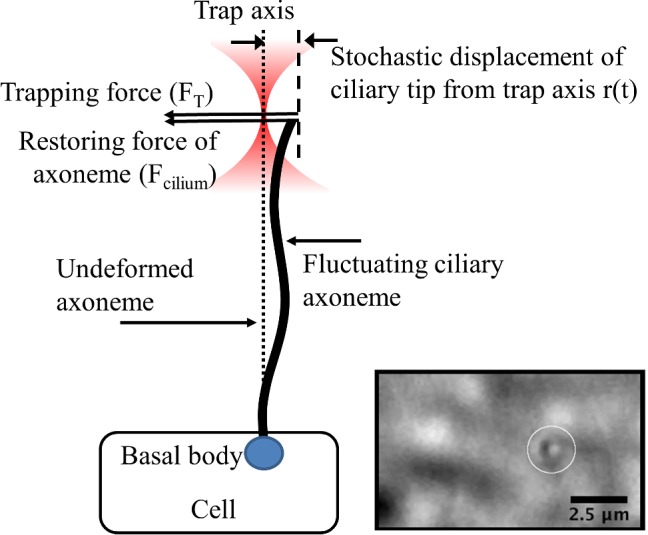
Schematic and inset image of optically trapped primary cilium. Location of optical trap indicated by circle. The cilium projects above the cell body and so appears as a dot in the microscopic image

A schematic and representative image of an optically trapped primary cilium is shown in Fig. 1. The cilium projects above the cell body, leaving the cells out of focus. As seen in the inset figure, the cilium appears as an in-focus dot against a blurred background.

**Fig. 2:**
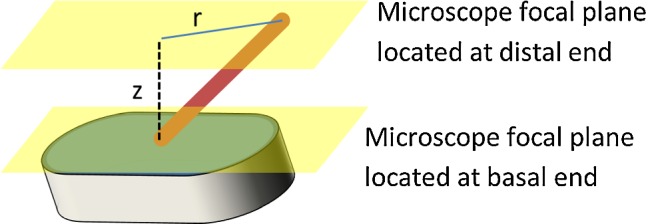
Schematic of cilium length measurement. Note, in practice cilia are not tilted so drastically and are nearly vertical

With the trap turned off, ciliated cells were located using brightfield imaging. The length of a cilium was measured optically by recording the *z*-distance required to move the in-focus object plane from basal end to distal end, and two images acquired to measure the projected distance ‘*r*’ between the basal attachment and distal tip. The cilium length $L = \sqrt{r^2 + z^2}$, see Fig. 2. The trap location within the field of view being previously located during the calibration step, the cilium distal tip was moved to the trap location. The trap was then turned on and QPD data acquired at 50 kSamples/s for several tens of seconds. Because the trap is applied only to the cilium tip and cilia are inextensible, the applied trapping force does not vary with cilium length. Furthermore, because the trapped cilium is observed simultaneously with brightfield illumination, we exclude the case of a bent or otherwise malformed cilium being trapped.

**Fig. 3:**
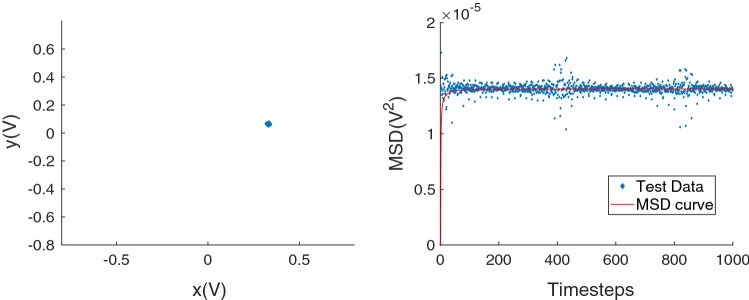
Sample subset of data acquired by the QPD, presented as position (left) and calculated MSD (right). *V* voltage

The tip of the cilium scatters the trapping beam, and as the tip moves stochastically due to constrained Brownian motion, the angular distribution of scattered light changes. QPD data consist of a time-series of voltage samples, say $V_i^j$, where $i = 1,2,3,4$ (each quadrant) and *j* is the sample number. For each sample, the four quadrants are combined to determine the location of the centroid along the ‘*x*’ and ‘*y*’ axis (axes are set by the rotational orientation of the QPD and fixed, but arbitrary with respect to the *x*- and *y*-axes of the camera), and the dataset $\left( j, V_x^j, V_y^j\right)$ is considered ‘raw data’. The time-series raw data are then processed to calculate the mean-squared displacement (MSD) of the distal tip, see Fig. 3. The scatter located in the regions $t = 0.008$ s and 0.017 s (400 and 800 timesteps, respectively) is an artifact. As we show, cilium length and asymptotic value of MSD are sufficient to characterize the mechanical response of a cilium in terms of a spring constant magenta $k_{\mathrm{cilium}}$.

A good overview of various optical trap analysis methods can be found in Jones et al. (2015). Our analysis method computes the MSD and fits an analytic function (presented below) to determine the long-time asymptotic value of the MSD, denoted ‘${\hbox {MSD}}_{\infty }$’. Analysis based on ${\hbox {MSD}}_{\infty }$ is preferred for two reasons. First, discretization of the QPD signal does not generate spurious results (Norrelykke and Flyvbjerg 2011). Second, precise knowledge of viscous damping, which is problematic as our trapped object is a slender cylinder and not a sphere, is not required.

### Calculation of trapped object MSD

Data analysis is done using a custom Matlab procedure ‘QPDanalysisBulk’ (Glaser et al. 2014) that uses time-series data collected by the quadrant photodiode (QPD), which measures the *x*- and *y*-positions of the centroid of light scattered by a trapped particle. The algorithm calculates the mean square displacement $\langle \Delta r^2 \left( \tau \right) \rangle$ from a trajectory *r*(*t*) discretized into $N_T + 1$ time steps consisting of ${N}_A = {N}_{T-\tau } + 1$ overlapping time intervals of duration $\tau$:

1 $$\begin{aligned} {{\text {MSD}}}^{{\mathrm{2D}}}\left( \tau \right) = \langle \Delta r^2 \left( \tau \right) \rangle = \frac{1}{N_A} \sum _{t=0}^{N_{T}-\tau } \left( r \left( t+\tau \right) -r\left( t \right) \right) ^2 \end{aligned}$$

By fitting ${\text {MSD}}^{\mathrm{2D}}$ to a known function (see subsections covering stochastic models), we obtained the long-time asymptotic limit value, which we denote ${\text {MSD}}_\infty ^{\mathrm{2D}}$.

### Structural model of a primary cilium

Following (Schwartz et al. 1997; Young et al. 2012; Downs et al. 2014; Resnick 2015), we initially modeled the cilium as a uniform cantilevered beam of length ‘*L*’ subject to external loading. This ‘heavy elastica’ model for a primary cilium treats the structure as a homogeneous isotropic flexible cylindrical beam with a hemispherical endcap, constrained at the basal end and free to move at the distal end. This model has been used for a wide variety of systems, including filamentous biopolymers (Wiggins et al. 1998), atomic force microscope tips (Sader 1998), and cellular protrusions including flagella (Lighthill 1976), stereocilia (Svrcek-Seiler et al. 1998), glycocalyx (Weinbaum et al. 2003), and actin brush border (Guo et al. 2000).

Because primary cilia, unlike flagella or motile cilia, do not actively generate internal forces, we may model the primary cilium as a passive elastic beam: there are no forces and/or moments generated within the beam. Because the slenderness (length/diameter) of the cilium is large, we may also neglect both rotatory inertia and transverse shear and approximately describe the ciliary axoneme shape in terms of a 1-D object, the so-called neutral axis (Mickey 1995).

In “Appendix” section, We present two variations of the heavy elastica model: the ‘classical’ cantilever and the ‘generalized’ cantilever. Compared to the classical model, the generalized cantilever has more degrees of freedom at the fixed end. The cilium spring constant ‘$k_{\mathrm{cilium}}$’ for each cantilever model is given by:

2 $$\begin{aligned} {\text {classical:}}\,k_{\mathrm{cilium}}= \frac{3 EI }{L^3} \end{aligned}$$

3 $$\begin{aligned} {\text {generalized:}}\,k_{\mathrm{cilium}}&=  \frac{3 EI J}{3 EI L+JL^3} \end{aligned}$$

where *J* is the rotational stiffness of the basal body, *L* is the cilium length, *E* is the Young’s modulus, *I* is the second moment of area, and *EI* is the bending rigidity of the cilium.

**Fig. 4:**
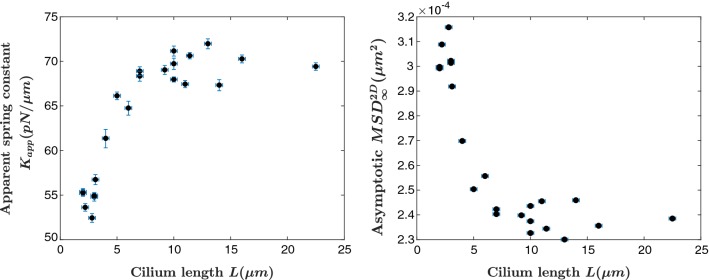
Calculated values of the apparent spring constant (left) and asymptotic MSD values of the trapped cilium tip (right) as a function of cilium length for untreated cilia

That is to say, if a cilium can be modeled as a homogeneous, isotropic material with bending rigidity *EI*, then our measurements should result in ${k}_{{\mathrm{cilium}}} \propto L^{-3}$. Our data (see Fig. 4) provide evidence that cilia do not mechanically behave as simple cantilevered beams.

### Stochastic model of an optically trapped cilium

Application of an optical trap to a cantilevered beam introduces at least two complications as compared to a trapped free particle. First, hydrodynamic (viscous) forces act along the entire length of the cantilever and not just the trapped end. Second, although the distal end of the cantilever can ‘freely’ respond to the applied trap force, the constrained end introduces a restoring force, embodied by the cilium spring constant $k_{\mathrm{cilium}}$. We denote the distance from the unbent cilium axis to the trap axis as ‘*d*’.

We first consider a simplified model of the trapped cilium—the axoneme is subject to viscous forces and behaves as an overdamped system, but the viscous damping coefficient $\gamma$ remains unspecified. Figure 1 represents this simplified model of the primary cilium, with the trap applied to the distal tip.

Considering 1D motion, from Fig. 1, we have a modified Langevin-type equation:

4 $$\begin{aligned} m {\ddot{r}}\left( t\right) = F_{\mathrm{trap}} + F_{\mathrm{cilium}} - \gamma \dot{r}\left( t\right) + \sqrt{2k_{\mathrm{B}}T \gamma } \, \zeta \left( t \right) \end{aligned}$$

where $F_{\mathrm{trap}} = - k_{\mathrm{trap}} r$, $F_{\mathrm{cilium}} =-k_{\mathrm{cilium}}\left( d + r \right)$, *m* is the mass, and $\gamma$ is the damping coefficient. $\zeta (t)$ is a normalized white-noise process, $k_{\mathrm{B}}$ is Boltzmann’s constant, and *T* the temperature.

Rearranging, we obtain our equation of motion:

5 $$\begin{aligned}&m {\ddot{r}} \left( t \right) + \gamma \dot{r} \left( t \right) + \left( k_{\mathrm{trap}} + k_{\mathrm{cilium}}\right) r \left( t\right) + k_{\mathrm{cilium}} \, d \nonumber \\&\quad =\sqrt{2k_{\mathrm{B}}T \gamma } \, \zeta \left( t \right) . \end{aligned}$$

Two key points to emphasize here are first, while ${k}_{\mathrm{trap}}$ should be the same for every trapped cilium, ${k}_{\mathrm{cilium}}$ may vary from cilium to cilium. Second, although the axoneme consists of 9 microtubule doublets, prior results demonstrate that the doublets are mechanically uncoupled (Battle et al. 2015). Furthermore, the flexural rigidity of each doublet is approximately twice that of a single microtubule. Taken together, our model assumes that the cilium spring constant is linearly proportional to the persistence length $l_{\mathrm{p}}$ for a single microtubule:

6 $$\begin{aligned} k_{\mathrm{cilium}} = 18 \left[ \frac{3 l_{\mathrm{p}} k_{\mathrm{B}} T}{L^3}\right] . \end{aligned}$$

Defining $k_{\mathrm{eff}} = k_{\mathrm{trap}}+ k_{\mathrm{cilium}}$, applying the Fourier transform to Eq. 5 to convert the displacement *r* to frequency domain in term of frequency $\omega$, we get

7 $$\begin{aligned} {\tilde{r}}(\omega )=\frac{{\tilde{F}}(\omega )}{m(\omega _0^2+i\omega \gamma /m-\omega ^2)} \end{aligned}$$

where $\omega _0^2=\frac{k_{\mathrm{eff}}}{m}$, ${\tilde{F}}(\omega )$ is the Fourier transform of external force and *i* is the imaginary number. The spectral density of the displacement is given as

8 $$\begin{aligned} {\varPhi }_r(\omega )=\frac{{\varPhi }_{\mathrm{F}}(\omega )}{m^2[(\omega _0^2-\omega ^2)^2 +\omega ^2\gamma ^2/m^2]} \end{aligned}$$

where ${\varPhi }_{\mathrm{F}}(\omega )$ is the spectral density of the external force. The 1D auto-correlation function (ACF) of the displacement is given as

9 $$\begin{aligned} {\text {ACF}}^{{\text {1-D}}}= \langle r(t) r(t+\tau )\rangle&=  \frac{1}{2\pi } \int _{-\infty }^{\infty } {\varPhi }_r(\omega ) e^{-i \omega \tau } {\text {d}} \omega \nonumber \\&=  \frac{1}{2\pi }\int _{-\infty }^{\infty } \frac{{\varPhi }_{{\rm F}}(\omega ) e^{-i \omega \tau } {\text {d}} \omega }{m^2[(\omega _0^2-\omega ^2)^2 +\omega ^2\gamma ^2/m^2]} \nonumber \\&=  \frac{k_{\mathrm{B}} T \gamma }{\pi m^2}\int _{-\infty }^{\infty } \frac{e^{-i \omega \tau } {\text {d}} \omega }{(\omega _0^2-\omega ^2)^2+\omega ^2\gamma ^2/m^2} \end{aligned}$$

since the spectral density of the noise is given as ${\varPhi }_{{\rm F}}(\omega )=2\gamma k_{\mathrm{B}} T$.

The above analysis is for 1D problems. In the case of a 2D problems, the auto-correlation function ${{\rm ACF}}^{{2\text{-}{\rm D}}}=2{{\rm ACF}}^{1\text{-D}}$. Applying the residue theorem to integrate Eq. 9 explicitly, in the 2D case, we get

10 $$\begin{aligned} {{\rm MSD}}^{{\rm 2D}}&=  \langle \Delta r^2(\tau )\rangle ^{{2\text{-}{\rm D}}} \nonumber \\&=  2[\langle r^2(0)\rangle -\langle r(\tau )r(0)\rangle ]^{{2\text{-}{\rm D}}} \nonumber \\&=  \frac{4 k_{\mathrm{B}} T}{k_{\mathrm{eff}}} \left[ 1 - e^{-\varGamma \tau /2} \left( \cosh \left[ \varOmega \tau \right] + \frac{\varGamma }{2 \varOmega } \sinh \left[ \varOmega \tau \right] \right) \right] \end{aligned}$$

where $\varGamma = \gamma /m$, $\varOmega ^2 = \frac{k_{\mathrm{eff}}}{m} - \varGamma ^2/4<0$ and the asymptotic limit as $\tau \rightarrow \infty$, ${\text {MSD}}^{\text {2D}}_{\infty } \equiv \frac{4k_{\mathrm{B}}T}{ k_{\mathrm{eff}}}$.

### Basal body fluctuations

We now include the role of basal body dynamics on ciliary motion (Battle et al. 2015) and determine what changes, if any, occurs in Eq. 10. Basal body fluctuations occur due to mechanical coupling of the basal body to actin-driven mechanical activity of the cell cortex. We now derive a simplified analytical model for an optically trapped cilium incorporating the induced fluctuations of the cilium tip caused by active fluctuations of the basal body. The underlying goal to refine Eq. 10 exists because our experimental apparatus determines the quantity ${\text {MSD}}^{\text {2D}}_{\infty }$.

The simplest way to incorporate stochastic motion of the basal body is to alter Eq. 5:

11 $$\begin{aligned}&m {\ddot{x}} \left( t \right) + \gamma {\dot{x}} \left( t \right) + \left( k_{\mathrm{trap}} + k_{\mathrm{cilium}}\right) x \left( t\right) \nonumber \\&\quad -\,k_{\mathrm{cilium}} \sqrt{\gamma {\mathrm{MSD}}_{\mathrm{bb}}/k_{\mathrm{bb}} } \zeta _1(t)= \sqrt{2k_{\mathrm{B}}T \gamma } \, \zeta _2 \left( t \right) \end{aligned}$$

where we have allowed the basal body to undergo active fluctuations with a mean-squared displacement $\mathrm{MSD}_{\mathrm{bb}}$ with an intrinsic spring constant $k_{\mathrm{bb}}$. We define their ratio as $\varTheta _{\mathrm{bb}}={\text {MSD}}_{\mathrm{bb}}/k_{\mathrm{bb}}$. We use two different (uncorrelated) white-noise processes $\zeta _1$ and $\zeta _2$. Going through the usual calculations, we obtain ${\text {MSD}}^{\text {2D}}_{\infty }$:

12 $$\begin{aligned} {\text {MSD}}^{\text {2D}}_{\infty } = \frac{4k_{\mathrm{B}} T}{k_{\mathrm{eff}}}+\frac{2 k_{\mathrm{cilium}}^2 \varTheta _{\mathrm{bb}}}{k_{\mathrm{eff}}} \end{aligned}$$

Finally, the apparent spring constant we got from the ${\text {MSD}}^{\text {2D}}$ is defined as

13 $$\begin{aligned} {K}_{\mathrm{app}}\equiv \frac{4 k_{\mathrm{B}} T}{{\text {MSD}}^{\text {2D}}_{\infty } }. \end{aligned}$$

Importantly, because $k_{\mathrm{cilium}}$ appears in both terms but ${\text {MSD}}_{\mathrm{bb}}$ in only one, there is a possibility to independently control $k_{\mathrm{cilium}}$ and ${\text {MSD}}_{\mathrm{bb}}$, providing multiple biochemical pathways to probe the ciliary flow response.

### Computational model

We developed a coarse-grained computational model of the primary cilium to study the deformation and fluctuations of the cilium axoneme and the basal body based on dissipative particle dynamics (DPD) (Peng et al. 2013). DPD is a coarse-grained molecular dynamics which aims to capture thermal fluctuations and hydrodynamic behaviors of the original atomistic systems (Groot and Warren 1997; Hoogerbrugge and Koelman 1992). Besides *non-bonded* DPD interactions to capture fluctuations and hydrodynamics, we apply coarse-graining to construct *bonded* interactions within the molecular structures of the primary cilium based on the current understanding of its structure. Details of the model are described in “Appendix”.

By applying our coarse-grained DPD model, we calculated the mean-squared displacement (MSD) of the tip held within an optical trap when the entire cell is within a hydrodynamic thermal bath consisting of DPD particles of water held at 37 $^{\circ }\hbox {C}$. We found that the MSD of the tip always increases with cilium length when we use either a constant persistence length for the microtubules or use the length-dependent persistence length from Eq. 16. Since we model the basal body as a rigid body described by the Langevin equation and model the remaining cellular components within the DPD framework, our model allows us to assign different temperatures for the Langevin equation (basal body) and the hydrodynamic bath. This approach, using a higher temperature for the basal body, models actin-driven “active fluctuations” of the basal body. Our model predicts the MSD of the tip decreases with the cilium length for either constant or length-dependent microtubule persistence lengths, which is qualitatively consistent with our experimental observations.

## Results

### Cilia have a length-dependent persistence length

Our experimental method directly obtains the long-time asymptotic value of the trapped cilium tip’s mean-squared displacement ${\text {MSD}}^{{\text {2D}}}_{\infty }$, which can more intuitively be presented as an apparent spring constant ${K}_{\mathrm{app}}$ for the trapped cilium tip, ${K}_{\mathrm{app}} \propto \frac{1}{{\text {MSD}}^{\text {2D}}_{\infty }}$. We present our obtained values of ${\text {MSD}}^{\text {2D}}_{\infty }$ and ${K}_{\mathrm{app}}$ as a function of cilium length in Fig. 4. Critically important is the fact that homogeneous cantilevered beams are characterized by a spring constant $k_{\mathrm{cilium}} \propto L^{-3}$, which is clearly not the case for cilia. As compared to homogeneous beams, cilia stiffens with increasing length.

### Improved analytical shell model of cilium structure

As our data shows, the ‘heavy elastica’ model (Schwartz et al. 1997) does not reproduce our results. Consequently, we now present a refined structural model of primary cilia. Rather than considering the ciliary axoneme as effectively homogeneous, following Liu et al. (2012) and Gao et al. (2010) we treat each axonemal microtubule doublet as transversely isotropic elastic shell with wall thickness $h = 2.7$ nm and middle radius $R = 12.5$ nm. The mechanics of our system can be parameterized by axial and azimuthal Young’s moduli $E_z$ and $E_{\theta }$, shear modulus *G*, Poisson ratio $\mu _z$, optical trap spring constant ‘$\hbox {k}_{\mathrm{trap}}$’ and the ratio ‘$\varTheta _{\mathrm{bb}}$’ from the stochastic motion of the basal body.

The essential result of this model (see “Appendix”) is that the persistence length $l_{\mathrm{p}}$ of each microtubule doublet depends on the aspect ratio $\alpha = L/R$:

14 $$\begin{aligned} l_{\mathrm{p}} (L)&=  {l_{{\mathrm{p}},\infty }}\big/ \left[ 1+\frac{\pi ^2}{\alpha ^2}\left( \frac{E_z}{G}-\frac{3}{2}\mu _z\right) +\frac{\pi ^4E_z}{2\alpha ^4 E_{\theta }} \right] \end{aligned}$$

15 $$\begin{aligned} l_{{\mathrm{p}},\infty }&=  \frac{\pi h R^3 E_z }{k_{\mathrm{B}} T} \end{aligned}$$

where *h* is the wall thickness of the microtubules, and *R* is the radius of the microtubules. Because, for microtubules, $\frac{E_z}{G}-\frac{3}{2}\mu _z \approx \frac{E_z}{G}$, we may use a simplified expression:

16 $$\begin{aligned} l_{\mathrm{p}} (L)={l_{\mathrm{p},\infty }}/ \left[ 1+\frac{\pi ^2}{\alpha ^2}\frac{E_z}{G} +\frac{\pi ^4E_z}{2\alpha ^4 E_{\theta }} \right] . \end{aligned}$$

### Parameter estimation

**Fig. 5:**
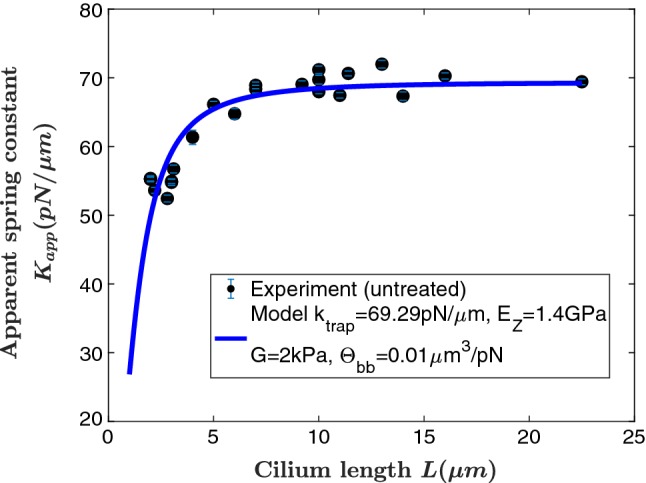
Experimental data and model fit curve for untreated cilia. Moduli values used here are $E_z = 1.4$ GPa, $E_{\varphi } = 2$ MPa, $G = 2$ kPa. Obtained best-fit parameter values are the optical trap spring constant $k_{\mathrm{trap}} = 69$ pN$/{\upmu }$m and $\varTheta _{\mathrm{bb}} = 0.01$ $\upmu {\mathrm{m}}^3/\mathrm{pN}$

Parameter estimations were obtained by fitting datasets to our model-derived expressions 6 and 12 using the nonlinear least-squared function ‘lsqnonlin’ in MatLab (MathWorks, Natick, MA). Confidence intervals of the fit parameters were also generated using MatLab. An example fit is shown in Fig. 5.

It is important to realize that this improved analytical model has few free parameters; various moduli of microtubules have been independently measured (Liu et al. 2012; Tuszynski et al. 2005; Memet et al. 2018; Kis et al. 2002, 2008; Pampaloni et al. 2006; Sept and MacKintosh 2010) with a variety of methods. Interestingly, while there is consensus for axial Young’s modulus $E_z$, there remains great uncertainty regarding values of the shear modulus *G*. We initially take $E_z = 1.4\,{\hbox {GPa}}$ as a representative value and leave $E_{\theta }$, G, ${k}_{\mathrm{trap}}$ and the ratio $\varTheta _{\mathrm{bb}}={\hbox {MSD}}_{\mathrm{bb}}$/$k_{\mathrm{bb}}$ as fit parameters.

### $E_{\theta }$ is not an essential parameter

Varying the parameter $E_{\theta }$ had no impact on the overall quality of fit, nor did the best-fit values for $k_{\mathrm{trap}}$ or $\varTheta _{\mathrm{bb}}$ change. Consequently, we conclude that $E_{\theta }$ is not an essential material parameter to model ciliary bending. Referring to Eq. 16, the parameter $E_{\theta }$ appears in a term associated with the (inverse) fourth power of cilium length and so has less effect as compared to the shear modulus *G*. It is also possible that the roughly isotropic distribution of microtubule doublets in the axoneme (ninefold symmetry) additionally reduces the role of $E_{\theta }$. A typical value we estimated for $E_{\theta }$ is 2 MPa.

### Estimate of shear modulus *G*

We find that our data can be fit with 95% confidence values of 1.6 kPa $< G < 2.4$ kPa, with a best-estimate $G = 2.0$ kPa. This value is in agreement with Pampaloni et al. (2006) and Gao et al. (2010) but is quite divergent as compared to Memet et al. (2018) and Kis et al. (2008). One possibility, as discussed in Memet et al. (2018), is that our measurement occurs in the “low-strain” regime, while reports of MPa and GPa values of *G* are obtained in “high-strain” experiments, typically involving buckling.

### Basal body fluctuations are required to model the data

If we set the basal body fluctuations equal to zero, we are unable to fit the data; thus, according to our model, basal body fluctuations are required to correctly model our data. Based on prior simulations (Sept and MacKintosh 2010) and measurements of the mechanical response of microtubules (Kis et al. 2008; Tuszynski et al. 2005), we set ${E}_z = 1.4$ GPa, ${E}_{\theta } = 2$ MPa and $G = 2$ kPa, resulting in best-fit parameter values $k_{\mathrm{trap}} = 69$ pN/${\upmu }$m as the optical trap spring constant (95% confidence interval [$67\,{\mathrm{pN}}/{\upmu }\hbox {m}$, 71 ${\mathrm{pN}}/{\upmu }\hbox {m}$]) and $\varTheta _{\mathrm{bb}}$ = 0.01 $\upmu \,{\mathrm{m}}^3/\mathrm{pN}$ as the ratio between the mean-squared displacement of the basal body and its intrinsic spring constant $k_{\mathrm{bb}}$. Our estimate for $\varTheta _{\mathrm{bb}}$ appears to be wholly novel, although evidence for the existence of ${\hbox {MSD}}_{\mathrm{bb}}$ appears in Battle et al. (2015).

### Pharmacological manipulation of cilia

**Fig. 6:**
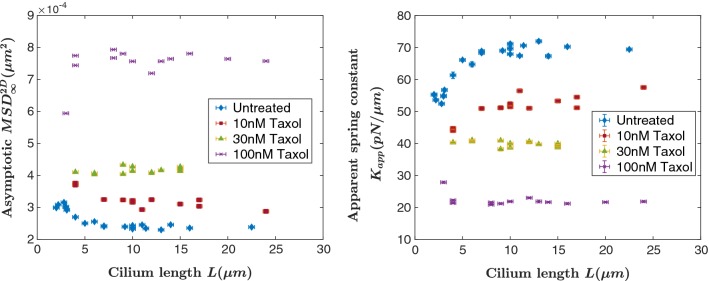
Calculated values of the apparent spring constant and asymptotic MSD values for taxol-treated cells as a function of cilium length

Taxol (paclitaxel) is a microtubule stabilizer, yet perhaps counterintuitively, prior studies applying Taxol to microtubules (Felgner et al. 1996) and cilia (Resnick 2016) show that the flexural rigidity decreases. Based on molecular dynamics simulations, it was proposed (Sept and MacKintosh 2010) that Taxol increases axonemal stability by allowing the increased flexibility to relieve internal stresses. We characterized the effect of Taxol on ciliary mechanics by adding low concentrations of Taxol to cell culture media for 24 h prior to trapping. Based on a dissociation constant ${K}_{\mathrm{d}} = 10$ nM (Caplow et al. 1994) we measured the effect of adding 10 nM, 30 nM, and 100 nM Taxol to cell culture media on ciliary mechanics. Figure 6 presents our data. Low concentrations of Taxol were used to minimize alterations to the microtubule cytoskeletal elements. We observed that at the low concentrations of Taxol used, cells were phenotypically unchanged.

### $E_z$ and *G* depend on Taxol concentration

As before, best-fits were very insensitive to changes in $E_{\theta }$. Attempts to fit the data by allowing the remaining parameters $k_{\mathrm{trap}}$, $\varTheta _{\mathrm{bb}}$, $E_z$ and *G* to simultaneously vary were not successful, so we obtained best-fit values by successive fitting to the data. First, best estimates of $k_{\mathrm{trap}}$ and *G* were obtained by first fixing the value of$\varTheta _{\mathrm{bb}}$ obtained for untreated cilia. Next, computed estimates of $k_{\mathrm{trap}}$ and *G* were used as inputs for a second fit, resulting in updated best-fit estimates of both $\varTheta _{\mathrm{bb}}$ and ${E}_z$. These ‘corrected’ values were then used as inputs to obtain updated fit values of $k_{\mathrm{trap}}$ and *G*, and the process iterated until all computed values converged to stable values.

The obtained estimates for $\varTheta _{\mathrm{bb}}$, ${E}_z$, and *G* are provided in Table 1. Our obtained values of taxol-treated ${E}_z$ are in agreement with previous measurements (Vinckier et al. 1996; Liu et al. 2012). Performing a Michaelis–Menten functional fit to ${E}_z$ as a function of Taxol concentration yields a dissociation constant ${K}_{\mathrm{d}} = 18$ nM, in good agreement with Caplow et al. (1994).

**Table 1:** Best-fit parameter values for $\varTheta _{\mathrm{bb}}$, $E_z$, and *G* as a function of Taxol concentration

Taxol concentration (nM)	$\varTheta _{\mathrm{bb}}$ ($\upmu \,\mathrm{m}^3/\mathrm{pN}$)	$E_z$ (GPa)	*G* (kPa)	$l_{{\mathrm{p}},\infty }$ (mm)
0	0.01	1.4	2.0	5.8
10	0.01	0.98	5.0	3.9
30	0.01	0.05	10.0	0.2
100	0.01	0.002	10.0	0.01

It should be noted that the difference in magnitudes between ${E}_z$ (GPa) and *G* (kPa) results in some numerical instability of the fitting procedure; our best-fit values of shear modulus *G* are thus limited in precision. Regardless, we see that the final best-fit values of $\varTheta _{\mathrm{bb}}$ are apparently Taxol-independent.

### Experimental error analysis

To demonstrate that our experimental results are valid, we now provide some error analysis. The errors in our best-fit values result from a combination of random and systematic errors; systematic error will be discussed first.

#### Measurement of trap stiffness

**Fig. 7:**
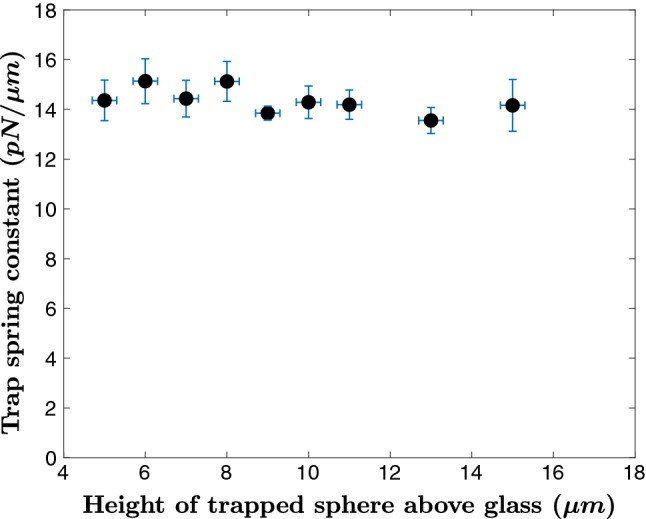
Apparent spring constant of optically trapped microspheres

We checked for any systematic error or variation in the measured trap stiffness as a function of trap height. This check was performed in case the QPD signal varied with optical path length. Using the microscope *z*-axis controller, we trapped microspheres at different heights above a glass slide and computed ${\text {MSD}}^{\text {2D}}_{\infty }$. Our data are shown in Fig. 7. Our data show that the trap stiffness varies very weakly with trap height and can be neglected. Thus, we have confidence that the length dependence of ciliary flexural rigidity is not an artifact of our apparatus.

We note there is no systematic error associated with trapping long vs. short cilia; for our trap, the beam waist is 0.3 $\upmu \hbox {m}$ and Rayleigh length is 0.4 $\upmu \hbox {m}$. Thus, the optical trap is essentially confined to a small volume that includes only the cilium tip. As cilia are inextensible, there is no axial free movement of cilia within the trap.

#### Sources of random error

The primary sources of independent random error are: measurement of cilium length and experiment-to-experiment variability of the trap stiffness $k_{\mathrm{trap}}$. The random error due to length measurement $\frac{\delta L}{L}$ varies with length because $\delta L$ is fixed by the objective lens, $\delta L = \pm 0.3\,\upmu \hbox {m}$. Uncertainties due to experimental variations in the trap stiffness are caused by the trap’s sensitive dependence on random variations of many parameters: optical alignment (including laser pointing stability), relative index of refraction between trapped object and surrounding fluid medium, diameter of primary cilium, potential mechanical disturbances to the optical bench, and so on. The collective trapping uncertainties simply result in random uncertainty in the calculated ${\text {MSD}}^{\text {2D}}_{\infty }$, which we directly observe as a result of QPD data processing. We estimate the total random error of the ${\text {MSD}}^{\text {2D}}_{\infty }$ to be on the order of 10%.

Another potential source of error is due to the ciliary axoneme being anchored within the cell body; a short segment of the cilium is within the cell body rather than the extracellular space and so is subject to a somewhat different environment. The magnitude of this error is difficult to rationally estimate but is expected to be small based on a discussion found in Downs et al. (2014).

A related potential source of error is the existence of a ‘ciliary pocket’ (Benmerah 2013), a feature resembling an endocytotic pit. It is not clear what impact on local flow dynamics, if any, the ciliary pocket would create. In other cases, the cilium does not fully emerge into the extracellular space. We would expect cilia that are partially or fully enclosed would have entirely different responses to extracellular flow. We note that our cultured cells did not present any evidence of enclosed cilia.

Cilia are not static structures; there is intraflagellar transport of not only structural proteins but also transmembrane proteins, all principally by the IFT488 complex; there could thus be some uncertainty in mechanical response due to the presence of these proteins as well as any axonemal–ciliary membrane attachments; this error is difficult to quantify but has likely been already accounted in the overall random error of ${\text {MSD}}^{\text {2D}}_{\infty }$. A prior report (Young et al. 2015) discussed this issue in some detail and indicates any effect is small.

### Alternative models of cilium structure

In this section, we provide summary results of several alternative (failed) approaches used attempt to explain the apparent length dependence of $k_{\mathrm{cilium}}$.

One potential solution may be to allow the bending modulus appearing in the Euler–Bernoulli equation to vary with position: $EI\,=\,EI(s)$. The Euler–Bernoulli equation itself is then modified. Unfortunately, the general equation that results from allowing $EI\,=\,EI(s)$ is very cumbersome and not obviously solvable. We thus present it as “Appendix.” Importantly, because the ciliary axoneme has a constant cross section and essentially a uniform structural composition along the length, it is unclear what could cause a spatially varying bending modulus. We do note that tubulin is subject to a variety of post-translational modifications that accumulate nonuniformly along the axoneme (Wloga et al. 2017), it is conceivable that these modifications could alter the axoneme stiffness in a spatially dependent manner; experiments to probe mechanical effects of post-translational tubulin modification have not yet been performed.

Next, we carefully examined the stochastic model for optically trapped objects immersed in a viscous medium as it applies to a cilium and focused specifically on viscous drag as a potential way to reconcile our experimental results with the use of a homogeneous beam model.

A lengthy calculation provided as “Appendix” shows that even when the drag coefficient $\gamma$ is correctly calculated for a cylinder of length ‘*L*’, there is no change to the form of ${\text {MSD}}^{\text {2D}}_{\infty }$. Our computational coarse-grained model further confirms that the hydrodynamic viscous force along the entire cilium can be equivalently represented as an effective force localized to the trapped end. Consequently, accounting for viscous drag does not “rescue” the homogeneous cantilever model.

Taken together, we conclude that our experimental results are not due to an experimental artifact, systematic error, or random error. Furthermore, neither incorporation of different boundary conditions, nor accounting for viscous drag, nor spatial variations in *EI* result in accurate modeling of our results.

## Discussion

We have found, for the first time, experimental evidence supporting a length-dependent mechanical model for primary cilia. The overall context of this work relates to how cells use cilia to sense fluid flow and how fluid flow can regulate cell and tissue function. Biological relevance lies in both diseases associated with abnormal cilia (ciliopathies) and also injury recovery (lack of fluid flow stimulus). It is possible that our data can help explain the persistent uncertainty in measured values of the ciliary flexural rigidity; in particular, the finding (Downs et al. 2014) that longer cilia apparently have a higher apparent bending modulus, in agreement with results presented here. Finally, we presented data demonstrating the impact of Taxol on the mechanical properties of cilia.

We have attempted to demonstrate that (1) in contrast to previous modeling efforts, cilia cannot be modeled as a homogeneous cantilever but rather as composed of a bundle of orthotropic shells, and (2) fluctuations of the basal body are an essential component of ciliary mechanics. We have demonstrated that our analytical method is well-matched to a class of experimental techniques that apply localized forces to the cilium, including optical trapping but also other related approaches (magnetic trapping, for example). We explored how the mechanical response of the microcantilever relates to underlying mechanical properties and identified the basal end of the primary cilium as a site of high interest, both because the structural properties are largely unknown but also because the basal body could be the site of initiation of mechanosensation responses.

Our results begin to address possible quantifiable relationships between (autoregulated) cilium length, biological responses to fluid flow, and the role of fluid flow in regulating cell and tissue function. Regarding our hypothesis that flexural rigidity may be involved in the regulation of cilium length, the existing literature is inconclusive. One issue is that in the absence of fluid flow stimulation, cilium length is often heterogeneous (Roth et al. 1988), and another is that fixation techniques damage the cilium (Mohieldin et al. 2015).

However, there is a known correlation between kidney injury and cilium length (Verghese et al. 2011). In this context of injury leading to localized ischemia-hypoxia, there is a tentative connection between increased cilium length and Hypoxia-Inducible Factor (HIF) stabilization (Verghese et al. 2011) and we have also published data showing that chemically stabilizing HIF results in more flexible cilia (Resnick 2016). Our hypothesis relating ciliary mechanics to ciliary length is reasonable. The work presented here should provide an increased ability to study our hypothesis, especially in the context of the Oak Ridge Polycystic Kidney (ORPK) (Lehman et al. 2008) mouse line, which expresses pathologically short cilia due to a mutation in the protein Polaris and is used as a model for polycystic kidney disease.

Another avenue for extension of our results lies with the boundary conditions at the basal end, that is, how to account for the basal body. A growing body of results (Hu and Nelson 2011; Lin et al. 2013; Battle et al. 2015; Resnick 2015) focuses on the basal body both as a modifier of the mechanical response and as some sort of ‘gate’ regulating the transport of materials in and out of the cilioplasm, thus the basal body potentially serves as the site of cellular flow sensing. We have demonstrated the possibility of investigating basal body dynamics by observing ciliary dynamics. In conclusion, our results provide a rational framework for the study of fluid flow sensing by primary cilia and identify potential therapeutic strategies to modify flow sensor sensitivity to restore normal physiological function.

## Figures and Tables

**Fig. 8:**
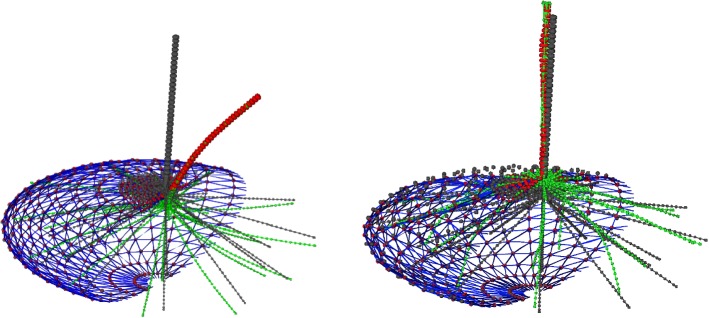
Coarse-grained simulations (only half of the cell membrane shown). Cilium shown in both untrapped/trapped configurations (left) and thermal fluctuations (right)

**Fig. 9:**
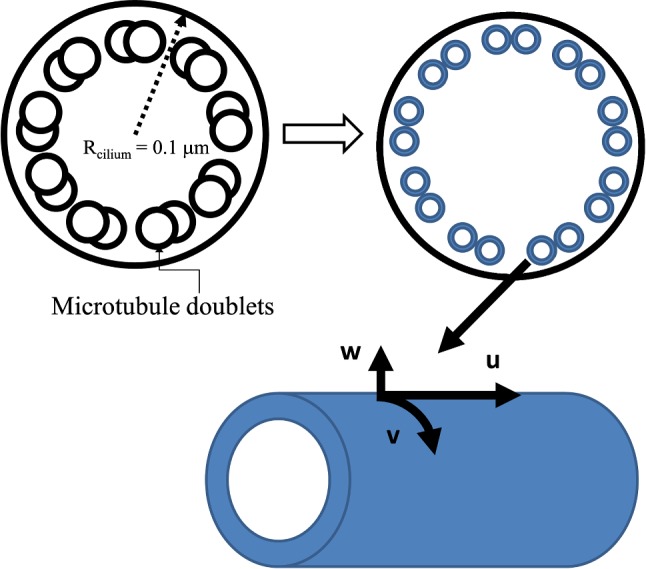
Transversely isotropic shell model of cilium structure. We consider the ciliary axoneme as consisting of 18 mechanically uncoupled microtubules, each modeled as an transversely isotropic shell with strains indicated

## Appendix: Continuum models of the primary cilium

### Classical cantilevered beam

The time-dependent shape *Y*(*s*, *t*) of the neutral axis of a cilium is given by the linearized Euler–Bernoulli law for pure bending (see for example, Rao 2007):

17 $$\begin{aligned} EI \frac{\partial ^{4} Y}{\partial s^{4}} + \mu \frac{\partial ^{2} Y }{\partial t^{2}}=q(s) \end{aligned}$$

where *q*(*s*) is an externally applied distributed load (force per length), ‘$\mu$’ is the mass per unit length, ‘*E*’ is the Young’s modulus, ‘*I*’ the area moment of inertia (for a cylinder of radius a, $I = \pi {a}^4/4$) and *EI* together referred to as the flexural rigidity or bending modulus, having units of force * area. Measurements of the flexural rigidity of primary cilia vary, but generally cluster between $EI =$ 10–50 ${\hbox {pN}}\,\upmu {\hbox {m}}^2$ (Battle et al. 2015; Downs et al. 2014; Schwartz et al. 1997; Young et al. 2012). We consider the cilium to be inextensible, and all deformations are considered transverse to the neutral axis. For simplicity, we reduce the full three-dimensional range of motion to deformations within a plane.

To solve the Euler–Bernoulli equation, we require boundary conditions for both the free and constrained ends. At the free (distal) end, the cilium responds to an applied load:

1. the bending moment vanishes:
  18 $$\begin{aligned} \frac{\partial ^{2} Y}{\partial s^{2}}\Big |_{s=L}=0. \end{aligned}$$
2. The free end is subject to shear from an applied end load, for example from the optical trap ‘$F_{\mathrm{T}}$’:
  19 $$\begin{aligned} \frac{\partial ^{3} Y}{\partial s^{3}}\Big |_{s=L}= \frac{-F_{\mathrm{T}}}{EI}. \end{aligned}$$
  and the constrained end has a ‘built in’ support:
3. The constrained end cannot move: Y(0)=0.
4. The constrained end is ‘built in’:
  20 $$\begin{aligned} \frac{\partial Y}{\partial s}\Big |_{s=0}=0. \end{aligned}$$

The classical cantilever has static solutions:

21 $$\begin{aligned} Y(s)&=\frac{F_{{\rm T}} (3L-s)s^2}{6 EI} \\ Y(L)&=\frac{L^3}{3 EI} F_{{\rm T}}. \\ \end{aligned}$$

#### Generalized cantilevered beam

Typically, the constrained end of cantilevered beams is classified as ‘free’, ‘clamped’ or ‘hinged’, depending on how the cantilever is attached to a substrate. The most general case of an end connected to a translational and rotatory spring, linear and torsional damper, and mass with rotational inertia is covered in Rao (2007). While boundary conditions for the basal attachment of cilia are not yet fully known, preliminary results (Battle et al. 2015; Resnick 2015; Young et al. 2012) have shown that the fixed end is neither free, clamped nor hinged. One current model treats the fixed end as constrained by a nonlinear rotatory spring obeying a force law ${F}(\theta ) = {J}\theta + \alpha \theta ^2$ (Young et al. 2012; Resnick 2015), where ‘*J*’ is the linear (Hookean) spring constant and ‘$\alpha$’ the nonlinear coefficient, while another incorporates a viscoelastic rotatory spring (Battle et al. 2015). The more general boundary condition replacing the fourth boundary condition (Eq. 20) is:

4. The constrained end is attached to a mass with rotational inertia $I_{\mathrm{m}}$ by a nonlinear torsional spring and linear torsional damper (coefficient ‘*c*’):
  22 $$\begin{aligned} \left[ \frac{\partial ^{2} Y}{\partial s^{2}} - \frac{L}{EI} \left( J \frac{\partial Y}{\partial s}+\alpha \left( \frac{\partial Y}{\partial s}\right) ^2 + c \frac{\partial ^2Y}{\partial s \partial t} +I_{\mathrm{m}}\frac{\partial ^3Y}{\partial s \partial t^2}\right) \right] _{s=0} =0. \end{aligned}$$

With this boundary condition, the static solution to the Euler–Bernoulli equation is found to be:

23 $$\begin{aligned} Y\left( s\right) = \frac{s\left[ s F_{{\rm T}} (3L-s)\alpha +3 EI(\sqrt{(J^{2}+4F_{{\rm T}}\alpha )}-J)\right] }{6 EI\alpha } \end{aligned}$$

24 $$\begin{aligned} Y\left( L\right) =\frac{L\left[ 2L^{2} F_{{\rm T}}\alpha +3 EI\left( \sqrt{\left( J^{2}+4F_{{\rm T}}\alpha \right) }-J\right) \right] }{6 EI\alpha }. \end{aligned}$$

The solution is constrained by requiring $L > 0$, $J > 0$, and $EI > 0$.

#### Beam bending equation with spatially varying bending modulus

For the linear theory of pure bending, points along the neutral line defined by the axial coordinate ‘*s*’ will be laterally deflected by an amount

25 $$\begin{aligned} y_{f}= \frac{M s^2}{2 EI} \end{aligned}$$

where *M* is the bending moment and *I* the ‘second moment of area’. If we allow $EI = EI(s)$, meaning the quantity EI may collectively vary with *s*, then along the neutral axis:

26 $$\begin{aligned} y'&=  \frac{2 (EI) Ms-Ms^2(EI)'}{2 EI}\\ y''&=  M\left[ \frac{2 (EI)-s^2 (EI)''}{2 (EI)^2}\right] \\ \end{aligned}$$

where we designate for example $\frac{{\mathrm{d}}y}{{\mathrm{d}}s}$, as $y^{\prime }$. Similarly, $y^{\prime \prime } \equiv \frac{{\mathrm{d}}^2 y}{{\mathrm{d}}^2 s}$. For static deflections generated by a distributed load *w*(*s*),

27 $$\begin{aligned} M^{\prime \prime } = w(s) \end{aligned}$$

which, after substitution results in:

28 $$\begin{aligned}w(s) &= \frac{\partial ^2 M}{\partial s^2} \\ \frac{\partial ^2 M}{\partial s^2} &= \frac{2 EI \left( EI^2 Y^{(4)} \left( s^2 EI (EI)^{\prime \prime }-2 \left( EI-s EI^{\prime }\right) ^2\right) ^2\right) }{\left( 2 \left( EI-s EI^{\prime }\right) ^2-s^2 EI(EI)^{\prime \prime }\right) ^3}\\&\quad +\,\frac{2 EI \left( 6 EI Y^{(3)} EI^{\prime } \left( s^2 EI (EI)^{\prime \prime }-2 \left( EI-s EI^{\prime }\right) ^2\right) ^2 \right) }{\left( 2 \left( EI-s EI^{\prime }\right) ^2-s^2 EI(EI)^{\prime \prime }\right) ^3}\\&\quad +\,\frac{2 EI \left( 3 EI (EI)^{\prime \prime } Y^{\prime \prime } \left( s^2 EI (EI)^{\prime \prime }-2 \left( EI-s EI^{\prime }\right) ^2\right) ^2+ 6 EI^{\prime 2} Y^{\prime \prime } \left( s^2 EI (EI)^{\prime \prime }-2 \left( EI-s EI^{\prime }\right) ^2\right) ^2\right) }{\left( 2 \left( EI-s EI^{\prime }\right) ^2-s^2 EI(EI)^{\prime \prime }\right) ^3}\\&\quad +\,\frac{2 EI \left( 2 s EI^2 Y^{(3)} \left( 2 \left( EI-s EI^{\prime }\right) ^2-s^2 EI (EI)^{\prime \prime }\right) \left( sEI (EI)^{(3)}+3 \left( 2 EI-s EI^{\prime }\right) EI^{\prime \prime }\right) \right) }{\left( 2 \left( EI-s EI^{\prime }\right) ^2-s^2 EI(EI)^{\prime \prime }\right) ^3}\\&\quad +\,\frac{2 EI\left( 2 s^2 EI^2 Y^{\prime \prime } \left( s EI(EI)^{(3)}+3 \left( 2 EI-s EI^{\prime }\right) EI^{\prime \prime }\right) ^2\right) }{\left( 2 \left( EIs EI^{\prime }\right) ^2-s^2 EI(EI)^{\prime \prime }\right) ^3}\\&\quad +\,\frac{2 EI\left( 6 s EI(EI)^{\prime } Y^{\prime \prime } \left( 2 \left( EI-s EI^{\prime }\right) ^2-s^2EI (EI)^{\prime \prime }\right) \left( s EI EI)^{(3)}+3 \left( 2 EI-s EI^{\prime }\right) EI^{\prime \prime }\right) \right) }{\left( 2 \left( EI-s EI^{\prime }\right) ^2-s^2 EI(EI)^{\prime \prime }\right) ^3}\\&\quad +\,\frac{2EI ^3 Y^{\prime \prime } (2 \left( EI-sEI^{\prime }\right) ^2-s^2 EI (EI)^{\prime \prime }) (EI (6 EI^{\prime \prime }+s (s EI^{(4)}+8 EI ^{(3)})))}{\left( 2 \left( EI-s EI^{\prime }\right) ^2-s^2 EI(EI)^{\prime \prime }\right) ^3}\\&\quad -\,\frac{2 EI \left( s^2 \left( 3 EI^{\prime \prime 2}+2 EI^{(3)} EI^{\prime }\right) \right) }{\left( 2 \left( EI-s EI^{\prime }\right) ^2-s^2 EI(EI)^{\prime \prime }\right) ^3}.\\ \end{aligned}$$

This expression reduces to the usual Euler–Bernoulli equation if EI is constant.

#### Exact results for a optically trapped homogeneous cantilever immersed in a viscous medium

Incorporation of hydrodynamic forces along the ciliary axoneme is complicated because the cilium is a slender structure. The approach (Sader 1998) used here considers the fluid drag to act as an (additional) effective mass.

Beginning with the Fourier transformed scaled time-dependent Euler–Bernoulli equation (Eq. 17), we account for hydrodynamic viscous drag $F_{\mathrm{Hydro}}$ by a fluid with density $\rho$, viscosity $\eta$, and an applied driving force $F_{\mathrm{d}}$ acting on a cantilever with (1-D) mass density ‘$\mu$’:

29 $$\begin{aligned}&\frac{EI}{L^4} \frac{\partial ^4 {\widetilde{Y}}(s,\omega )}{\partial s^4} - \mu \omega ^2 {\widetilde{Y}}(s,\omega ) ={\widetilde{F}}_{\mathrm{Hydro}} +{\widetilde{F}}_{\mathrm{d}} \nonumber \\&\quad {\widetilde{F}}_{\mathrm{Hydro}} =\left[ \omega \eta {{{Re}}} \varXi \left( {{{Re}}} \right) +3i\pi \eta a \omega \delta \left( L-s\right) \right] {\widetilde{Y}}(s,\omega ) \end{aligned}$$

*Re* is the Reynolds number $Re = \frac{\omega a^2\rho }{\eta }$. For primary cilia, $Re \ll 1$. The hydrodynamic drag terms correspond to flow around a cylindrical axoneme and flow around the hemispherical tip, respectively.

When the cylinder axis is perpendicular to the fluid velocity, the first terms of the hydrodynamic drag coefficient $\varXi \left( {{Re}} \right)$ are given by (Van Dyke 1975):

30 $$\begin{aligned} \varXi \left( Re\right)&=\frac{4\pi }{{Re}}\left[ \varDelta -0.87\varDelta ^{3}+O(\varDelta ^{4})\right] \\ \varDelta&= \left[ \frac{1}{2}-\xi -\ln \left( \frac{{Re}}{4}\right) \right] ^{-1} \end{aligned}$$

where $\xi$ is Euler’s constant ($\xi = 0.577\ldots$). The hydrodynamic drag force $F_{\mathrm{Hydro}} \rightarrow 0$ as Re $\rightarrow 0$, but importantly, the drag coefficient diverges: $\varXi \left( {{Re}} \right) \rightarrow {Re }^{-1}$ as Re $\rightarrow 0$.

The driving force $F_d$ consists of two terms: Brownian forces $F_{\mathrm{B}}$ and the optical trap ${F}_{\mathrm{trap}}$. We assume the optical trap applies a force only to the tip of the cilium $F_{\mathrm{trap}}=F_0\delta (s-L)$. For our trap, the beam waist is 0.3 $\upmu$m and Reyleigh length is 0.4 $\upmu$m, justifying the use of a localized applied force.

Constructing the eigenfunction mode expansion (index ‘*n*’) of the cantilever (Huang 1964)

$$\begin{aligned} {\widetilde{Y}}(s,\omega )&=\sum a_n(\omega )\phi _n(s)\\ \phi _n(s)&= A_n \cos(\beta s)+B_n \sin (\beta s) + C_n \cosh (\beta s)\\&\quad + D_n \sinh (\beta s)\\ \beta ^4&= \frac{\mu \omega ^2}{EI}\\ a_n(\omega )&= \left( \frac{L^4}{EI}\frac{1}{C_n^4 - B(\omega )^4}\right) \int _{0}^{1}\phi _n(s) {\widetilde{F}}_{{\mathrm{d}}}(s) {\mathrm{d}}s \\&= \left( \frac{L^3}{EI}\frac{1}{C_n^4 - B(\omega )^4}\right) \left( {\widetilde{F}}_{{\mathrm{B}}}+{\widetilde{F}}_{\mathrm{trap}}\right) \\ \end{aligned}$$

where ${\widetilde{F}}_{{\rm B}}$ is the Brownian force integrated along the cilium length and ${\widetilde{F}}_{\mathrm{trap}}$ the trap force, which is localized to the cilium tip:

$$\begin{aligned} {\widetilde{F}}_{{\mathrm{B}}}&= L\int _{0}^{1}\phi _n (s){\widetilde{F}}_{{\mathrm{B}}}(s) \\ {\widetilde{F}}_{\mathrm{trap}}&= \phi _n (1)F_0 \\ \end{aligned}$$

We also defined

$$\begin{aligned} B(\omega )&=C_n\left( \frac{\omega }{\omega _1}\right) ^{1/2}\left( 1+\frac{\pi \rho a^2}{4\mu }\varXi \left( Re\right) +\frac{i3\eta a}{\mu \omega }\delta (s-L)\right) ^{1/4}\\ \end{aligned}$$

$C_n = 1.875, 4.694, 7.854, 10.995\ldots$. are roots of the eigenvalue characteristic equation and $\omega _1 = 3.51 \sqrt{\frac{EI}{\mu L^4}}$ is the lowest eigenvalue for oscillation in an inviscid medium. For additional details, please see Sader (1998).

The eigenmode expansion simplifies considerably for trapped cilia when evaluated at the distal tip. In this case, a single mode ($n = 1$) is dominant and the Brownian force is uncorrelated with the (static) trap force:

$$\begin{aligned} Y(L,\omega )&= \left( \frac{L^3}{EI}\frac{1}{C_1^4 - B(\omega )^4}\right) \phi _1(1)\left( {\widetilde{F}}_{{\mathrm{B}}}+{\widetilde{{\mathrm{F}}}}_{\mathrm{trap}}\right) \\ \left| Y(L,\omega )\right| ^2&= \left| \frac{L^3}{EI}\frac{2}{C_1^4 - B(\omega )^4}\right| ^2\left( 4k_{\mathrm{B}} T\gamma (\omega )+4 F^2_0\right) \\ \end{aligned}$$

where $\gamma (\omega )$ is the damping coefficient for which we obtain an explicit expression. After a considerable amount of algebra, we obtain:

$$\begin{aligned} \left| Y(L,\omega )\right| ^2&= \frac{{\mathrm{2D}}\varGamma ^2}{\left[ \left[ \omega _{\mathrm{cilium}}^2-\omega ^2\right] ^2+\omega ^2\varGamma ^2\right] ^2}\\ D&= k_{\mathrm{B}}T/\gamma \\ \varGamma&= \gamma / m\\ \omega _{\mathrm{cilium}}^2&=\frac{\omega _1^2}{\alpha }\\ \gamma&=\frac{3\pi \eta }{ \alpha }L\\ \alpha&=1+\frac{\pi \rho a^2}{4\mu }\varXi \left( Re\right) \\ \end{aligned}$$

The MSD is obtained by Fourier transforming $\left| Y(L,\omega )\right| ^2$ and we obtain the same MSD expression provided in Eq. 10. Therefore, including viscous drag effects on a slender cantilever model for a cilium does not ‘rescue’ the homogeneous cantilever model.

### Setup of the computational coarse-grained model of the primary cilium

As shown in Fig. 8, we explicitly model the nine vertical microtubule doublets of the axoneme (covered by cilia membrane) and cytoplasmic microtubules (green) connecting basal body to the actin cortex (blue) and the lipid-bilayer (red). The basal body including the centrioles and surrounding pericentriolar material (PCM) was modeled as a rigid sphere, which can rotate under the constraints from connecting microtubules. The microtubules in the axoneme and the cytoplasmic microtubules are connected to the basal body as a clamped boundary condition. More importantly, the actin cortex and its interaction with microtubules were also explicitly modeled. The lipid-bilayer (red) and the actin cortex (blue) are modeled as two distinct components that couple together, with a two-dimensional triangulated network for the cortex. Details of this model can be found in Peng et al. (2013).

### Governing equation of transversely isotropic cylindrical shell

We model the cilium axoneme as a transversely isotropic material. In the cylindrical coordinate system (${r},\theta ,{z}$), the normal strains $\varepsilon _{z}, \varepsilon _{\theta }$ and shear strain $\varepsilon _{z \theta }$ of the shell in the isotropic ${z}-{\theta }$ plane are related to the displacement fields as (refer to Liu et al. 2012)

31 $$\begin{aligned} {\varepsilon }=\left\{ \begin{array}{l} \varepsilon _{z} \\ \varepsilon _{\theta }\\ \varepsilon _{z \theta } \end{array} \right\} =\left\{ \begin{array}{ll} \frac{\partial u}{\partial z}-r \frac{\partial ^2 w}{\partial z^2} \\ \frac{\partial v}{\partial \theta } +\frac{w}{R+r}-\frac{Rr}{R+r} \frac{\partial ^2 w}{\partial \theta ^2} \\ \frac{R}{R+r} \frac{\partial u}{ \partial \theta }+\frac{R+r}{R}\frac{\partial v}{\partial z}-\frac{2Rr+r^2}{R+r} \frac{\partial ^2 w}{\partial z \partial \theta } \end{array} \right\} , \end{aligned}$$

where $u(z,\theta ),v(z,\theta ), w(z,\theta )$ are displacements in $z, \theta , r$ directions, see Fig. 9. For transversely isotropic linear elastic materials, the stresses are related to the strains as

32 $$\begin{aligned} \left[ \begin{array}{lll} \sigma _{z} \\ \sigma _{\theta }\\ \tau _{z\theta } \end{array} \right] =\left[ \begin{array}{lll} {E_z}/{(1-\mu _{\theta }\mu _z)} &{}\quad { \mu _{\theta } E_z}/{(1-\mu _z\mu _{\theta })} &{}\quad 0 \\ {\mu _z E_{\theta }}/{(1-\mu _z\mu _{\theta })} &{}\quad {E_\theta }/{(1-\mu _{\theta }\mu _z)} &{}\quad 0 \\ 0 &{}\quad 0 &{}\quad G \end{array} \right] \left[ \begin{array}{lll} \varepsilon _{z} \\ \varepsilon _{\theta }\\ \varepsilon _{z\theta } \end{array} \right] , \end{aligned}$$

where $\mu _{\beta }$ are the Poisson ratios along the direction ‘$\beta$’, $E_{\beta }$ the Young’s moduli ($E_z \mu _{\theta } =E_{\theta }\mu _z)$, and *G* the shear modulus. The material symmetry reduces the number of independent stiffness (or compliance) coefficients from 9 to 5, and as a consequence Poisson ratios are presented with a single subscript rather than two, see for example Ding et al. (2006, page 18). The stress resultants are related to the stresses and displacement fields; referring to Liu et al. (2012), we may obtain expressions for the internal normal and shear forces and moments of a shell element undergoing small displacements.

After a lengthy calculation, we obtain the contour-length-dependent persistence length when a single bending mode dominates (${n}=1$):

33 $$\begin{aligned} l_{\mathrm{p}}(L)&=  {l_{{\mathrm{p}},\infty }}\big/ \left[ 1+\frac{\pi ^2}{\alpha ^2}\left( \frac{E_z}{G}-\frac{3}{2}\mu _z\right) +\frac{\pi ^4E_z}{2\alpha ^4 E_{\theta }} \right] \end{aligned}$$

34 $$\begin{aligned} l_{{\mathrm{p}},\infty }&=  \frac{\pi h R^3 E_z}{k_{\mathrm{B}} T} \end{aligned}$$

where $$\alpha =L/R$$.
